# Strategies for the Removal of Polysaccharides from Biorefinery Lignins: Process Optimization and Techno Economic Evaluation

**DOI:** 10.3390/molecules26113324

**Published:** 2021-06-01

**Authors:** Sandra Corderi, Tom Renders, Kelly Servaes, Karolien Vanbroekhoven, Tony De Roo, Kathy Elst

**Affiliations:** 1 Flemish Institute for Technological Research (VITO), Separation & Conversion Technology, Boeretang 200, 2400 Mol, Belgium; sandra.corderigandara@vito.be (S.C.); Kelly.Servaes@vito.be (K.S.); karolien.vanbroekhoven@vito.be (K.V.); 2 INEOS Phenol Belgium NV, Haven 1930, Geslecht 1, 9130 Beveren, Belgium; tom.renders@ineos.com (T.R.); Tony.De.Roo@ineos.com (T.D.R.)

**Keywords:** hydrolysis lignin, polysaccharides, purification, characterization, biorefinery, TEA, acid hydrolysis, precipitation

## Abstract

The utilization of biorefinery lignins as a renewable resource for the production of bio-based chemicals and materials remain a challenge because of the high polysaccharide content of this variety of lignins. This study provides two simple methods; (i) the alkaline hydrolysis-acid precipitation method and (ii) the acid hydrolysis method for the removal of polysaccharides from polymeric biorefinery lignin samples. Both purification strategies are optimized for two different hardwood hydrolysis lignins, HL1 and HL2, containing 15.1% and 10.1% of polysaccharides, respectively. The treated lignins are characterized by polysaccharide content, molecular weight, hydroxyl content, and Attenuated Total Reflection-Fourier Transform Infrared Spectroscopy (ATR-FTIR). Preliminary techno-economic calculations are also carried out for both purification processes to assess the economic potential of these technologies. The results indicate that both protocols could be used for the purification of HL1 and HL2 hydrolysis lignins because of the minimal polysaccharide content obtained in the treated lignins. Nevertheless, from an industrial and economic perspective the acid hydrolysis technology using low acid concentrations and high temperatures is favored over the alkaline hydrolysis-acid precipitation strategy.

## 1. Introduction

Research towards alternative and renewable resources to substitute fossil fuels has increased in recent years. Lignocellulosic biomass and especially lignin, an underutilized phenolic component of biomass, is considered a bio-based building block for chemical applications. Lignocellulosic biomass is comprised of three main polymeric components: cellulose, hemicellulose, and lignin. Lignin is the second most abundant renewable biopolymer on earth, after cellulose, and accounts for 20–30% of lignocellulosic biomass [1].

In contrast to cellulose and hemicellulose, lignin is a phenolic heteropolymer consisting of *p*-hydroxyphenyl (H), guaiacyl (G), and syringyl (S) monomer building blocks, which are linked irregularly through ether and carbon–carbon bonds. Softwood lignin is almost exclusively built of G units and hardwood lignin contains both S and G units in ratios typically varying from 1:1 to 3:1. Grass lignin consists of H, G, and S units, with H units usually being less abundant than G and S units [2].

Lignin is closely linked to cellulose and hemicellulose contributing to the mechanical strength of the plant cell wall. Lignocellulose processing takes place in biorefineries, which usually involves biomass fractionation into carbohydrate and lignin-derived product streams. Most biorefineries focus mainly on the valorization of cellulose and hemicellulose for the production of high-value products, while lignin is considered a low-value byproduct [3]. The most abundant source of lignin is the paper and pulp industry. Kraft and sulfite pulping are the two dominating process technologies used in chemical pulping, generating about 50 million tons of lignin per year out of which 1.65 million tons were commercially available in 2018 [4]. Despite the high-value opportunities, Kraft lignin is primarily burned for energy production, while sulfite pulping lignin (i.e., lignosulfonate) is commercialized as raw material for low-value products such as industrial detergents, binders, dyes, concrete additives, or plasticizers [5]. On the other hand, the commercial production of organosolv, soda, and hydrolysis lignins is becoming significant and accounts for 75,000 tons per year [4]. Organosolv lignin is mainly produced at laboratory scale or pilot scale and it is not currently commercially available. Soda lignin is produced in sulfur-free soda pulping processes and is used for production of thermoset additives, animal health, and nutrition [6].

Hydrolysis lignins are primarily obtained after enzymatic and/or acid treatments of lignocellulosic biomass in biorefineries. The cellulosic ethanol industry, with an estimated production ratio of 0.5 kg lignin per kg of ethanol, is probably one of the largest sources of hydrolysis lignin, which is predominantly incinerated on site for energy recuperation [7,8]. The following unit operations are typically involved in the lignocellulosic ethanol production: feed handling and size reduction, pretreatment by dilute acid, enzymatic hydrolysis, fermentation, ethanol, and lignin separation [7]. Several acid treatments with sulfuric acid [9] and phosphoric acid [10] have also been used for the fractionation of lignocellulosic biomass and yields hydrolysis lignins.

Despite the challenges, which are (i) the structural complexity and heterogeneity of lignin, (ii) the variability of biomass sources and treatment processes, and (iii) the presence of impurities [11], the number of publications about lignin isolation, purification, fractionation, chemical modification, and potential applications has grown exponentially in the recent years [4,12,13]. These applications include binders [14], surfactants [15], dispersants for cement [16], carbon fibers [17,18], epoxy, polyurethane and phenol formaldehyde resins [19,20,21,22], thermoplastic elastomers [23], and fire retardants [13] among others. Given the applicability in diverse segments, lignin valorization could play an essential role in the bio-based economy and will contribute to the mitigation of climate change [24].

The (bio-)chemical applications of lignin usually require well defined molecular weight, functionality, reactivity, and homogeneity. These properties can be improved by depolymerization, fractionation, and the purification of lignin sources. Depolymerization reactions such as acid/base, reductive/oxidative, enzyme-catalyzed, and thermal treatments (pyrolysis and gasification) target the production of small(er) molecules such as monomers and/or oligomers [3]. On the other hand, membrane filtration, precipitation by pH switch, or extraction using green solvents are commonly applied lignin fractionation techniques for the improved valorization of polymeric lignins. Both chemical and physical treatments cause changes in lignin in terms of structure, molecular weight distribution, functionality, and impurities.

Hydrolysis lignins are usually associated with residual carbohydrates (cellulose and hemicellulose), which vary from 5% to 30% by weight [25] depending on the lignin isolation method and also depending on the post-treatment process. These polysaccharide residues are covalently linked to lignin which renders it challenging to obtain pure lignin. Furthermore, these polysaccharides can have a major effect on lignin properties such as solubility or reactivity. In order to unleash the full valorization potential of this lignin family, the removal of carbohydrates is an important step to enhance lignin quality and its application scope. Several lignin purification methods such as extraction with green solvents, membrane separation, treatment with ionic liquids, alkaline hydrolysis-acid precipitation, and ultrasonic extraction were reported in literature [26,27,28,29]. As a result, ash, free carbohydrates, sulfur content, and the molecular weight of lignins could be reduced. However, to the best of our knowledge, these processes have not been applied to the challenging removal of polysaccharides linked to hydrolysis lignins.

In addition, evaluation of techno-economic feasibility is an important aspect in process development but the evaluation has not yet been reported to our knowledge for hydrolysis type lignin purification processes. A techno-economic analysis is typically based on a discounted cash flow methodology to determine profitability measures such as internal rate of return (IRR) and pay-back time [30,31]. In addition, this type of analysis requires a good understanding of both the investment cost and annual cash flows. These economic figures often involve high uncertainties (too high) in very early development stages. As a first stepping stone toward such a techno-economic analysis, a *back-of-the-envelope* comparison of the major operational costs and income per ton product can already provide valuable information. Operational costs should obviously not exceed expected income, which is a first economic feasibility criterium. Moreover, this type of analysis allows the identification of the major cost contributors, which can guide further process development. In this article, two purification methods for the removal of polysaccharides from hydrolysis lignins, (i) alkaline hydrolysis followed by acid precipitation and (ii) acid hydrolysis, are proposed and the effects of these purification strategies on the lignin properties are investigated. These processes were selected because of their simplicity and their demonstrable capability for such purification. The efficiency of these processes for the removal of polysaccharides is evaluated by using two hydrolysis lignins derived from different biorefinery processes containing up to 15% polysaccharides. Optimal experimental conditions are established for each purification method and lignin source. The effects of process parameters on the characteristics of lignins (polysaccharide content, molecular weight, and OH content) and the comparison of these characteristics before and after the purification treatments and in between the studied purification protocols are discussed. In addition, ATR-FTIR analysis technology is applied to characterize lignin and to quickly estimate the presence of carbohydrates in the lignin samples. Furthermore, in order to evaluate industrial viability, the techno-economic competitiveness of the products obtained after both purification strategies is assessed.

## 2. Results and Discussion

### 2.1. Alkaline Hydrolysis-Acid Precipitation Treatment

In this study, the alkaline hydrolysis-acid precipitation treatment is used with focus on the reduction in the polysaccharide content in the selected hydrolysis lignin samples. The alkaline hydrolysis-acid precipitation treatment was previously described in literature for the purification of Kraft and Organosolv lignin. As a result of the treatment, ash, sulfur, carbohydrate content, and the average molecular weight (*M*_w_) of lignins were considerably reduced [12,18,29,32] and their solubility in organic solvents were improved [33].

The purification process leads to the production of two lignin fractions. An insoluble residue (IR) after the alkaline treatment and a soluble fraction, which is recovered by acid precipitation and washing of the precipitate for the removal of salts. This precipitate is designated as purified lignin, PL. The effect of NaOH concentration, temperature, and initial concentration of lignin in alkaline water (*W*_lign_) on the composition of IR and PL after the alkaline hydrolysis-acid precipitation treatment is investigated for the studied lignins. The initial concentration *W*_lign_ is expressed in weight % and determined by considering the dry matter of the starting materials, HL1 and HL2, and the mass of the NaOH solution. Moreover, the effects of this purification treatment on the chemical structure of the lignins are determined by polysaccharide content, *M*_w_, OH content (aliphatic, aromatic, and carboxylic acid), and FTIR measurements. The *M*_w_ and OH content could only be measured for the purified lignin fraction because of the poor solubility of the insoluble residues in the GPC and ^31^P-NMR solvents.

**Figure 1:**
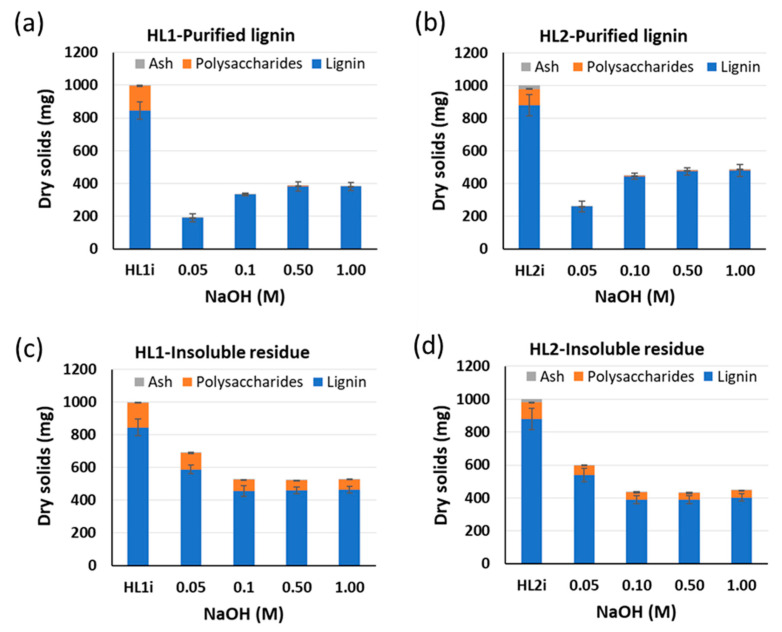
Influence of NaOH concentration on the composition of purified lignin (PL) for (**a**) HL1 and (**b**) HL2 lignins and insoluble residue (IR) for (**c**) HL1 and (**d**) HL2 lignins. Experiments are using initial *W*_lign_ = 0.03 and *T* = 25 °C. HL1i and HL2i represent the initial lignins before treatment. Standard deviation error bars of duplicate experiments.

In Figure 1, the influence of the NaOH concentration on the composition (lignin, carbohydrates, and ash content) of PL and IR fractions for HL1 and HL2 is shown. In general, the amount of lignin in PL shows an opposite trend to the amount of lignin in IR. When comparing HL1 and HL2 lignins under the same conditions, the ratio of PL to IR is higher for HL2 than HL1 and, as consequence, a higher amount of purified lignin for HL2 than for HL1 is obtained. The total yield of the purified lignin fractions ranges from 19 to 45 wt% for HL1 and from 26 to 50 wt% for HL2. For the same initial *W*_lign_, a slight increase in the amount of purified lignin fraction is observed when increasing the NaOH concentration. The increase is especially significant when moving from 0.05 M to 0.1 M NaOH for both lignins. High yields of purified lignin at increased NaOH concentrations were also described by L. Zoia et al. [32] in the purification of wheat straw lignin from bioethanol production. Usually, lignins dissolve very well at alkaline pH due to the ionization of its weakly acidic phenolic and carboxyl groups. These groups will tend to deprotonate less with decreasing NaOH concentration and, thus, the existing electrostatic interactions will decrease. This will lead to self-aggregation of the lignin macromolecules causing precipitation and, consequently, the reduction in lignin solubility [34].

On the other hand, the amount of polysaccharides remaining in the insoluble residues is much higher (60–101 mg and 42–59 mg for HL1 and HL2, respectively) than in the purified lignin fractions, which contain less than 5 mg polysaccharides for both lignins. The low amount of polysaccharides (<5 mg), corresponds with a concentration inferior to 1% d.m. for all the purified lignin fractions. The measured ash content for the initial lignins and their PL and IR fractions is minimal. The polysaccharide-to-lignin ratio, defined as the mass of polysaccharides in each of the fractions divided by the respective lignin mass (calculated by subtracting carbohydrates and ash from the mass of the obtained fractions), is also determined for each of the samples. The polysaccharide-to-lignin ratios of 0.3–0.7% and 0.7–1.0% for HL1 and HL2 purified lignin fractions, respectively, are obtained. As for the insoluble residue fractions, the polysaccharide-to-lignin ratios of 12.9–17.2% and 10.4–15.8% for HL1 and HL2, respectively, are obtained. Hence, the alkaline hydrolysis-acid precipitation treatment could be considered as a combination of both hydrolysis and fractionation processes, where a purified lignin fraction with negligible polysaccharide content is separated from an insoluble residue fraction that still contains polysaccharides. No appreciable effect of the NaOH concentration on the polysaccharide content of the purified lignin fractions is observed. Regarding the insoluble residues, a slight decrease in the polysaccharide content with increasing NaOH concentration is obtained and the effect is more visible in the case of HL1 than HL2 lignins.

**Figure 2:**
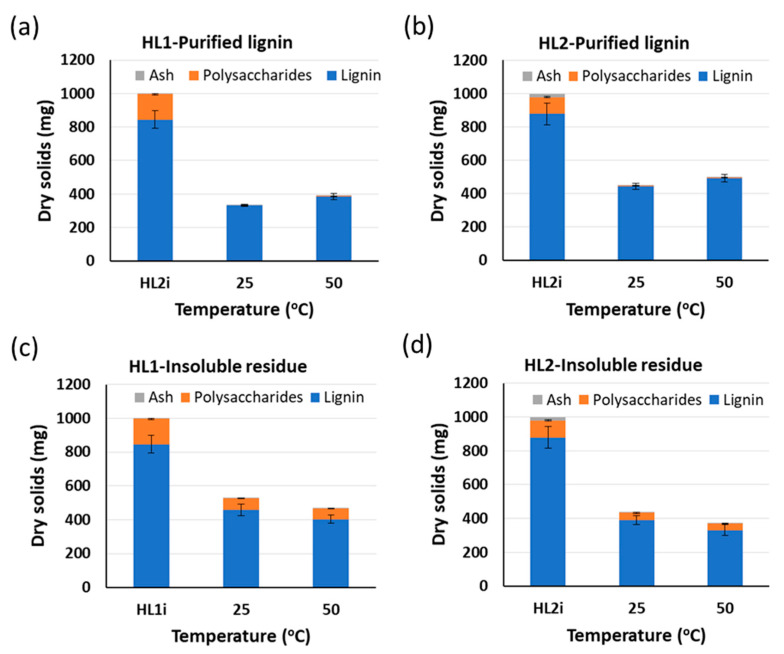
Influence of alkaline hydrolysis temperature on the composition of purified lignin (PL) for (**a**) HL1 and (**b**) HL2 lignins and insoluble residue (IR) for (**c**) HL1 and (**d**) HL2 lignins. Experiments are using 0.1 M NaOH and initial *W*_lign_ = 0.03. HL1i and HL2i represent the initial lignins before the treatment. Standard deviation error bars of duplicate experiments.

The influences of the alkaline hydrolysis temperature on the composition (lignin, carbohydrates, and ash content) of purified lignin and insoluble residue fractions are presented in Figure 2. The amount of lignin in the PL fraction increases with the temperature for both lignin sources due to the solubility enhancement of the lignins at elevated temperatures [32,34]. On the contrary, the amount of lignin present in the IR fraction decreases with the temperature. The amount of polysaccharides is much higher in the IR than in the PL fraction and it slightly increases with higher temperature for both HL1 (PL) and HL2 (PL).

**Figure 3:**
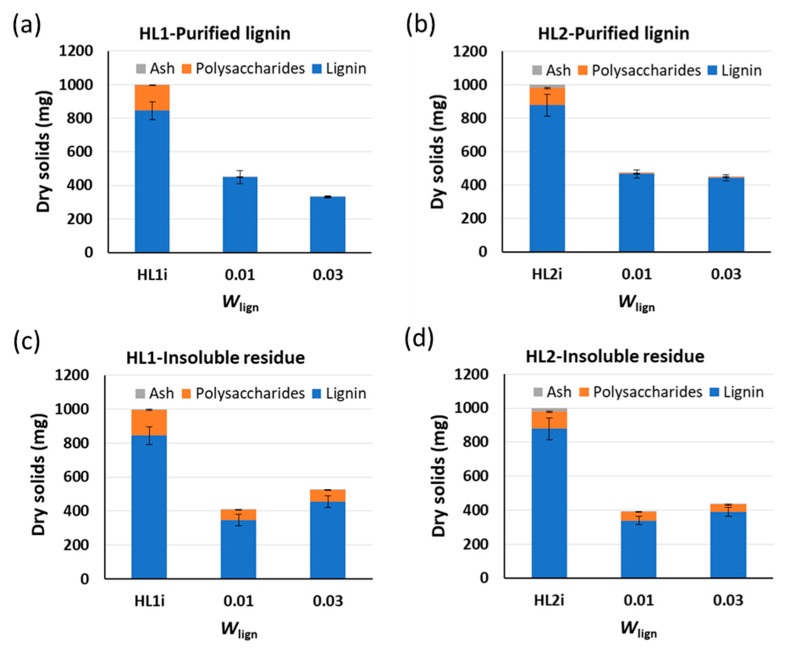
Influence of concentration of starting material (in weight %) in alkaline water on the composition of purified lignin (PL) for (**a**) HL1 and (**b**) HL2 lignins and insoluble residue (IR) for (**c**) HL1 and (**d**) HL2 lignins. Experiments are using 0.1 M NaOH and T = 25 °C. HL1i and HL2i represent the initial lignins before treatment. Standard deviation error bars of duplicate experiments.

The impact of the concentration of the starting material (in weight %) in alkaline water on the composition (lignin, carbohydrates, and ash content) of PL and IR fractions for HL1 and HL2 is shown in Figure 3. The amount of lignin in PL and IR fractions is influenced by the initial weight % of HL1 lignin and shows a decrease in HL1 lignin solubility and hereby a lower amount of HL1 lignin in PL when increasing the initial weight % of this lignin. This effect is minimal in the case of HL2 lignin. Concerning the remaining amount of polysaccharides in both PL fractions after the treatment, it is observed that a decrease in the initial weight % of lignin leads to a slight increase in polysaccharide content in both fractions. 

**Figure 4:**
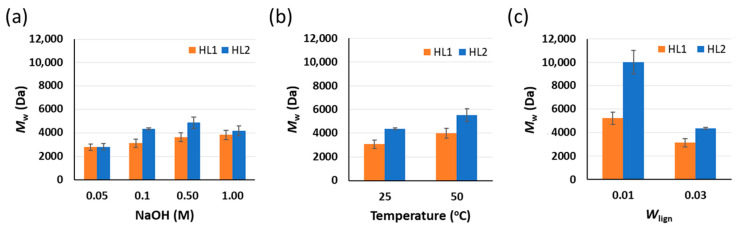
Influence of (**a**) NaOH concentration (T = 25 °C, initial *W*_lign_ = 0.03); (**b**) temperature (0.1 M NaOH, initial *W*_lign_ = 0.03); and (**c**) initial weight % lignin (0.1 M NaOH, T = 25 °C) on the average *M*_w_ of the obtained purified lignin (PL) fractions for HL1 and HL2 lignins. Standard deviation error bars of duplicate experiments.

The *M*_w_ of the purified lignin fractions subjected to different NaOH concentrations, temperature, and initial *W*_lign_ during the alkaline hydrolysis-acid precipitation treatment is depicted in Figure 4. The starting materials and the insoluble residues are not soluble in the GPC solvent (0.1M NaOH aqueous solution) and their *M*_w_ could not be determined. All purified lignin samples are fully soluble in the GPC solvent and, in all cases, the HL1 purified lignin fraction shows lower *M*_w_ than the HL2 purified fraction. In general, the yield of PL correlates with its *M*_w_: the higher the yield of PL, the higher its *M*_w_. Since the yield of PL is directly related to the amount of solubilized lignin during the alkaline hydrolysis, an increase in this amount indicates that higher *M*_w_ oligomers become solubilized, which will lead to an increase in the *M*_w_ of the purified lignin fractions. The *M*_w_ slightly increases with the NaOH concentration for HL1 and in the case of HL2 reaches a plateau around 4000 Da. Moreover, an increase in the alkaline hydrolysis temperature leads to an increase in the PL fraction *M*_w_. Finally, the effect of the initial *W*_lign_ on the *M*_w_ of purified lignin fractions is very pronounced and is observed to strongly increase the *M*_w_ of PL when decreasing the initial *W*_lign_. Therefore, it could be concluded that after the alkaline hydrolysis-acid precipitation treatment the lowest *M*_w_ fractions of PL are obtained when decreasing NaOH concentration and temperature and increasing the initial *W*_lign_.

The total OH content together with the aliphatic, aromatic, and carboxylic acid hydroxylic contributions of the purified lignin fractions obtained via alkaline hydrolysis-acid precipitation were measured by ^31^P-NMR and the results are presented in Table 1. Under similar treatment conditions, the total OH concentration of PL fractions is higher for HL1 than HL2 lignin, especially due to the contribution of the aromatic OH concentration, which is much higher for HL1 than HL2. However, the concentration of aliphatic OH was higher for HL2 than HL1. When comparing both lignins under the same experimental conditions, the highest yields and *M*_w_ fractions of PL were obtained for the HL2 lignin, while the highest total OH content, with large aromatic contributions, was observed for HL1 lignin.

In general, the same trend regarding total OH is observed when comparing the purified lignin fractions of HL1 and HL2 for varying NaOH concentration. The total OH content of the purified lignin fractions increases with the concentration of NaOH due to the increase in the aromatic OH contribution. Regarding the aliphatic OH concentration, similar values are observed when moving from 0.1 M to 1 M NaOH for both lignins. The total OH content decreases with the increase in temperature for HL1 and remains stable in the case of HL2. The decrease in the initial *W*_lign_ of HL1 leads to higher total OH content of this lignin because of the increase in aliphatic, aromatic, and carboxylic acid hydroxyl contributions. In general, high carboxyl or phenolic hydroxyl groups on lignins are related to a high number of reactive sites available for chemical modifications and related to an improved solubility in organic solvents [35]. As a result, the use of lignins in bio-based applications could be boosted.

ATR-FTIR spectroscopy is used for the fast characterization of the lignin samples after the alkaline hydrolysis-acid precipitation and acid hydrolysis purification treatments. The removal of polysaccharides from lignin can be observed in the FTIR spectra. Table S1 (supplementary information) summarizes the assignment of common absorption bands of lignin samples and Table S2 (supplementary information) includes the modified FTIR lignin bands after the removal of polysaccharides and their assignments according to the literature data.

**Figure 5:**
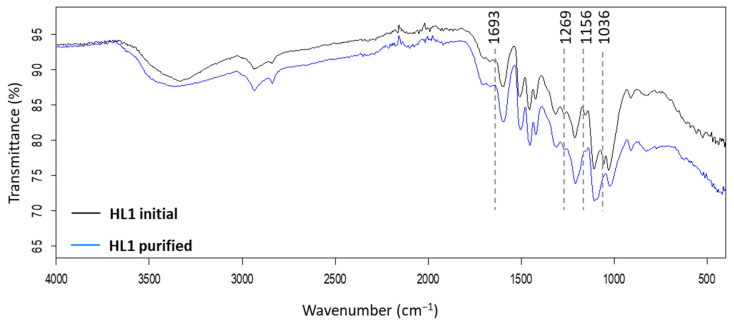
FTIR spectra of the initial HL1 and purified fraction after the alkaline hydrolysis-acid precipitation using 0.1 M NaOH, initial W_lign_ = 0.03, and T = 25 °C. Highlighted bands correspond to polysaccharides.

As an example, Figure 5 shows the FTIR spectra of the initial HL1 compared to its purified fraction via alkaline hydrolysis-acid precipitation using 0.1 M NaOH, initial *W*_lign_ = 0.03, and T = 25 °C. The carbohydrate bands are indicated in the figure. The signals at 1693 cm^−1^ and 1269 cm^−1^ are assigned to the C=O stretching in carbohydrates [36,37], the signal at 1156 cm^−1^ to the C-O stretching in ester groups in carbohydrates [38], and the 1036 cm^−1^ to the stretching vibration of ether bond in polysaccharide [39]. In this case, a reduction is observed of the 1036 cm^−1^ and 1156 cm^−1^ bands in the purified lignin fraction compared to initial HL1 sample, which corresponds to the elimination of polysaccharides after the alkaline hydrolysis-acid precipitation purification treatment. The FTIR spectra of initial HL1 and HL2 lignins, purified lignins, and insoluble residues after the alkaline hydrolysis-acid precipitation treatment are shown in Figure S1a,b (supplementary information). The FTIR spectra of purified lignins, obtained under different NaOH concentrations, temperatures, or initial mass fraction of lignin are observed in Figure S2 (supplementary information).

### 2.2. Acid Hydrolysis Treatment

An acid hydrolysis strategy is also explored for the removal of polysaccharides from the HL1 and HL2 hydrolysis lignin samples. Acid hydrolysis is primarily described in literature for the fractionation of biomass into lignin and polysaccharide fractions [40] but has been scarcely investigated in the context of lignin purification [9]. In this study, we investigate the efficiency of a one-step acid hydrolysis treatment for the reduction in polysaccharide content in the lignin samples. More specifically, the lignin samples are hydrolyzed in a treatment with H_2_SO_4_ at different concentrations, temperatures, and times and the effects of these parameters on the purity of the obtained lignins is determined. In contrast to the alkaline hydrolysis-acid precipitation treatment, only one lignin fraction is obtained after the acid hydrolysis treatment and this fraction is denoted as purified lignin (PL). The yield of PL after the acid hydrolysis treatment is ≥95% for every experiment. The polysaccharide concentration in the PL samples after the acid hydrolysis is measured for all the samples. The *M*_w_ is also determined for the samples which are soluble in the GPC solvent. Unfortunately, the OH content of the PL samples could not be determined because of their insolubility in the ^31^P-NMR solvent.

**Figure 6:**
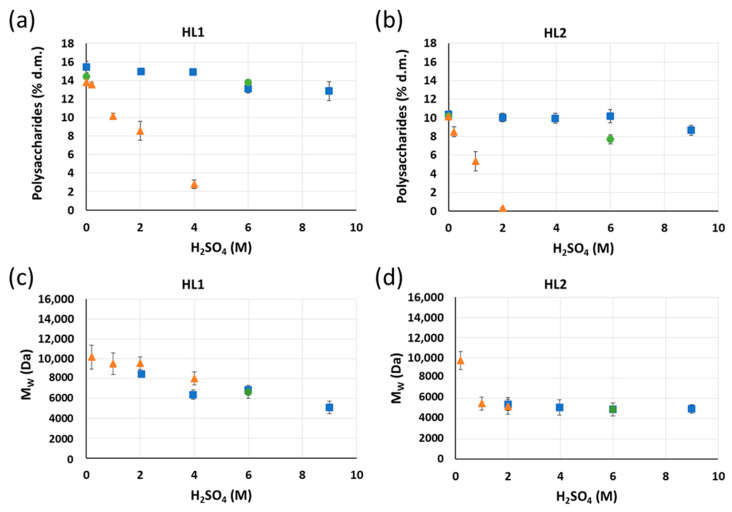
Concentration of polysaccharides in (**a**) HL1 and (**b**) HL2 and average M_w_ of (**c**) HL1 and (**d**) HL2 lignins after 1 h of acid hydrolysis treatment at different H_2_SO_4_ concentrations and temperatures (■ 30 °C; **●** 70 °C; 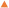 120 °C). Yields of purified lignins ≥ 95%. Standard deviation error bars of duplicate experiments.

In this case, the removal of polysaccharides is attributed to the cleavage of the covalent lignin–polysaccharides bonds by acid hydrolysis. Figure 6a,b depicts the concentration of polysaccharides in the HL1 and HL2 lignins, respectively, after the acid hydrolysis treatment as a function of the sulfuric acid concentration and temperature.

An increase in the acid concentration leads to a decrease in the polysaccharide content in the HL1 and HL2 samples and this effect is especially pronounced when increasing the temperature. When comparing HL1 and HL2, it is observed that harsher conditions are needed to purify HL1 than HL2. For example, a HL1 purified lignin sample with 2.8% polysaccharide content and a HL2 purified lignin sample containing 0.3% polysaccharides are obtained using 4 M and 2 M H_2_SO_4_ at 120 °C, respectively. This finding could be associated with the higher initial carbohydrate content of HL1 (15.1%) compared to HL2 (10.1%), which would mean that more lignin–polysaccharide covalent bonds would need to be broken in HL1 than in HL2 and, therefore, more acid would be needed to achieve a similar result.

The effect of the one-step acid hydrolysis treatment in HL1 and HL2 lignin samples is also investigated by GPC and the results are shown in Figure 6c,d for HL1 and HL2, respectively. The average *M*_w_ of HL1 and HL2 lignin samples after the acid hydrolysis processing is influenced by the acid hydrolysis conditions. The increase in acid concentration leads to a decrease in the *M*_w_ of HL1 and HL2 lignins. A competition between the cleavage of lignin–lignin and lignin–polysaccharide bonds and lignin condensation reactions likely occurs during the acid hydrolysis treatment of HL1 and HL2 lignins. This treatment not only breaks the lignin–polysaccharide bonds but also could hydrolyze the lignin by cleaving its inter-unit bonds, thereby reducing its molecular weight. Additionally, condensation reactions after the acid hydrolysis could also occur, resulting in an increase in the lignin average *M*_w_.

**Figure 7:**
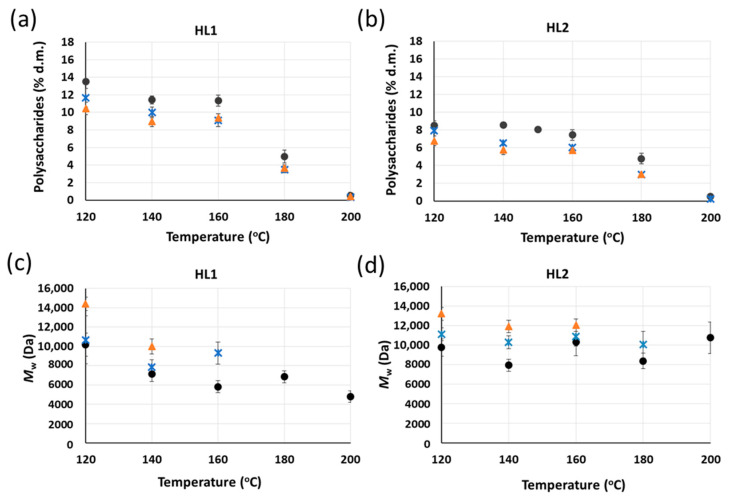
Concentration of polysaccharides in (**a**) HL1 and (**b**) HL2 and average M_w_ of (**c**) HL1 and (**d**) HL2 lignins after acid hydrolysis treatment using 0.2 M H_2_SO_4_ at different temperatures and time (● 1 h; 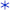 6 h; 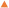 22 h). Yields of purified lignins ≥ 95%. Standard deviation error bars of duplicate experiments.

The purification of these lignins via acid hydrolysis using 2–4 M H_2_SO_4_ at 120 °C could be achieved. However, from an industrial and sustainability point of view, these concentrations could be considered excessively high. In order to obtain a sustainable and economically feasible acid hydrolysis treatment, low acid concentrations are mandatory and, in this regard, 0.2 M is set as the maximum acid concentration. Taking into consideration that an increase in temperature is beneficial for the acid hydrolysis purification treatment, experiments using 0.2 M H_2_SO_4_ at high temperatures (120–200 °C) and times (1–22 h) are carried out for HL1 and HL2. As shown in Figure 7a,b, the use of low acid concentrations for the acid hydrolysis could be compensated by the use of high temperatures and times for the reason that the higher the temperature and time of acid hydrolysis, the lower the polysaccharide content in HL1 and HL2 samples.

Moreover, it is observed that a temperature of at least 180 °C is needed to reduce the polysaccharide content in both lignins lower than 5% using 0.2 M H_2_SO_4_. When the temperature is increased from 160 °C to 180 °C, a sharp decrease in the polysaccharide content in both lignins is observed, although this effect is more pronounced for HL1 than for HL2 lignin. This finding highlights the strong impact of temperature on the removal of polysaccharides from HL1 and HL2 lignins via acid hydrolysis using low acid concentration (0.2 M H_2_SO_4_). The increase in time, at a constant temperature, from 1 h to 6 h and from 6 h to 22 h, during the acid hydrolysis treatment using low acid concentration also results in a decrease in the polysaccharide concentrations in HL1 and HL2 lignin samples. Nevertheless, the decrease in the polysaccharide content is largest when moving from 1 h to 6 h than from 6 to 22 h, which could indicate the reaching of a plateau in the polysaccharide removal over time.

In Figure 7c,d, the *M*_w_ of HL1 and HL2 lignins after the acid hydrolysis treatment using 0.2 M H_2_SO_4_ at different temperatures and time is displayed. Only the *M*_w_ results for the samples fully soluble in the GPC solvent are displayed in these figures. It is shown that an increase in the acid hydrolysis temperature, at a constant time and using low acid concentration (0.2M H_2_SO_4_), causes a decrease in the *M*_w_ of HL1, although some exceptions are observed at 160 °C and 180 °C. Regarding HL2, an unclear trend is observed when changing temperature and the *M*_w_ values range from 8000 Da to 13,000 Da. On the other hand, an increase in *M*_w_ of HL1 and HL2 is observed upon increasing the reaction time at a constant temperature. Therefore, long reaction times of the acid hydrolysis treatment of HL1 and HL2 lignins could promote lignin condensation reactions over depolymerization.

FTIR measurements of the lignin samples after the acid hydrolysis treatment are also carried out and the results can be found in the supplementary information (Figures S3–S5).

### 2.3. Assessment of Industrial Viability

#### 2.3.1. Alkaline Hydrolysis-Acid Precipitation

**Figure 8:**
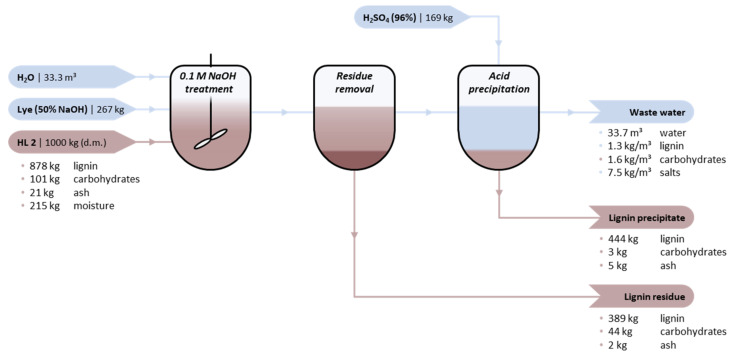
Simplified mass flow scheme for the batch alkaline hydrolysis-acid precipitation of 1000 kg HL2. Washing steps are not included for simplicity. Process parameters: 0.1 M NaOH, W_lign_ = 0.03, and T = 25 °C.

In order to evaluate potential industrial viability, a theoretical exercise is made based on the required input and output to treat 1000 kg (d.m.) of either HL1 or HL2. Figure 8 summarizes the results for the alkaline hydrolysis-acid precipitation method of HL2. The conditions selected are 0.1 M NaOH, 25°C, and *W*_Lign_ of 0.03. Based on the experimentally investigated conditions, these conditions provide the most optimal balance between high lignin precipitate yield and low chemicals/energy consumption (see Section 2.1).

The lignin concentration (*W*_Lign_ of 0.03) implies a water consumption of 33 m^3^ per 1000 kg HL2. In order to achieve a concentration of 0.1 M NaOH, 267 kg of lye (50 wt%) is required. After the treatment, 169 kg of H_2_SO_4_ (96 wt%) is required to induce precipitation, based on the stoichiometric neutralisation of NaOH. The yield of the targeted lignin precipitate is 452 kg based on lab scale experiments, 98 wt% (d.m.) of which is comprised of lignin. The carbohydrate-containing lignin residue amounts to 435 kg, which still has a carbohydrate content of 10 wt% (d.m.). In addition, there is a third output stream which constitutes the waste water (33.7 m^3^). The composition of this stream is theoretically approximated based on mass balance calculations. The waste water comprises approximately 54 kg carbohydrates (or derivatives thereof), 45 kg soluble lignin, and 249 kg salts; the concentrations are presented in Figure 8. The respective chemical oxygen demand (COD) was estimated to theoretically [41] equal to 146 kg. It is envisioned that treatment of this waste water would at least be comprise of two stages: A tailored primary treatment is required to make the waste water suitable for final processing in a more conventional and secondary treatment (e.g., aerobic oxidation). The specific primary treatment is considered to be inside the battery limit, while the secondary treatment could be outside. Designing and selecting a suitable primary treatment requires further research.

In addition, a back-of-the-envelope calculation is made comparing the costs of chemicals and feedstock with potential revenues, both for HL1 and HL2 (Table 2). Taking into consideration the above-mentioned experimental parameters, the total cost of feedstock, chemicals, and secondary waste water treatment (€428–438) approximately compensates the potential revenues (€446–541). Hence, the alkaline hydrolysis-acid precipitation method does not seem to be profitable at this stage. An important element that compromises industrial viability is the high water-to-lignin ratio (i.e., 33:1), which in itself is costly due to the high consumption of water. In addition, the dilute lignin:water mixture implies high amounts of NaOH to achieve the targeted concentration (0.1 M) and in turn high amounts of H_2_SO_4_ to eventually neutralize the base. Increasing the lignin-to-water (*W*_Lig_) ratio and decreasing the NaOH concentration are potential strategies to reduce costs. However, these adjustments conflict with earlier observed trends (Section 2.1) and will cause a substantial decrease in the lignin precipitate yield, thereby also reducing potential revenues. Overall, the consumption of chemicals and generation of waste are too high for the amount of the purified product.

#### 2.3.2. Acid Hydrolysis

**Figure 9:**
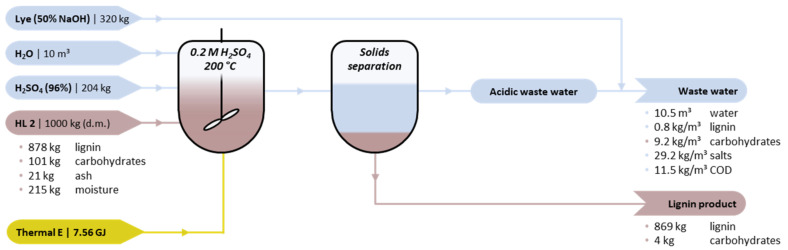
Simplified mass flow scheme for the acid hydrolysis of 1000 kg HL2. Washing steps are not included for simplicity. Process parameters: 0.2 M H_2_SO_4_, W_lign_ = 0.1, and T = 200 °C.

A similar theoretical exercise is made to treat 1000 kg HL2 using acid hydrolysis (Figure 9). A sulfuric acid concentration of 0.2 M is chosen in combination with a temperature of 200 °C. From the experimentally tested conditions, these result in the lowest possible acid consumption combined with the extensive removal of carbohydrates. Nevertheless, the consumption of concentrated sulfuric acid is very high (204 kg/1000 kg HL2) given that the lignin-to-water ratio is 10 wt%. Consequently, the process requires 320 kg lye (50% NaOH) to neutralize the waste water. The waste water, furthermore, contains a very high organic load (120 kg COD), requiring a primary treatment as part of the battery limit followed by a secondary treatment, which is similar to the alkaline hydrolysis-acid precipitation (vide infra). 

The magnitude of acid and base consumption is similar to that of the alkaline hydrolysis-acid precipitation method. An important advantage of acid hydrolysis is that the majority of the lignin is recovered as valuable product. The product comprises 869 kg lignin (assuming 99% recovery) and 4 kg carbohydrates. Ash content was not measured and therefore not included in the mass balance. A disadvantage of acid hydrolysis is that a high temperature is required (200 °C) for effective carbohydrate removal, while alkaline hydrolysis is feasible at ambient temperature. The required thermal energy to heat 10 m^3^ of slurry to 200 °C is estimated at 7.56 GJ, assuming no heat integration. Taking into consideration the process of heating with steam from a natural gas steam boiler operating at 90% efficiency, the natural gas intake required equals 9.30 GJ/ton HL2 or 2.58 MWh (higher heating value). In addition, the acid hydrolysis at 200 °C under a sufficiently high pressure requires high-quality materials to prevent corrosion.

An analogous back-of-the-envelope cost exercise is made, as presented in Table 3. For the acid hydrolysis, potential revenues (€837–869) are higher than the envisioned costs of feedstock, chemicals, and energy (€449–439) given the unit cost assumptions indicated in Table 4. The positive balance is mainly driven by the substantially higher lignin product yield and, to a smaller extent, by the lower water consumption compared to the alkaline hydrolysis, even though the consumption of acid and base is somewhat higher. Hence, the acid hydrolysis looks more promising than the alkaline hydrolysis-acid precipitation.

Notwithstanding, results should be interpreted with absolute care since more information is required to rigorously evaluate profitability. For example, capital investment and other variable costs have not yet been taken into account. Capital expenses are expected to be higher for the acid hydrolysis method as a result of the corrosive environment (H_2_SO_4_ and 200 °C) and autogenous water pressure (*ca.* 15 bar) compared to the alkaline system operating under ambient temperature. In addition, the composition and treatment of the waste water is another important yet unknown factor. For every 1000 kg HL treated, 100 kg to 150 kg of carbohydrates is brought into solution. It is expected that substantial carbohydrate degradation (i.e., humification) occurs during the high temperature acidic treatment, which would hamper any further carbohydrate valorization. The removal of the organic content (i.e., primary waste water treatment) is required prior to discharge and will add a substantial cost depending on the method. Different methods are available, including physico-chemical and biological treatments [45].

## 3. Materials and Methods

### 3.1. Chemicals

Two biorefinery hydrolysis lignins were studied in this work. HL1 was obtained from a biorefinery process using phosphoric acid and mixed hardwood biomass. HL2 was isolated from hardwood (birch) by thermo-chemo-mechanical extrusion. The properties of these hydrolysis lignins are summarized in Table 4. These properties were measured following the protocols described in the analytical methods section.

These lignins contain very low ash, Na or S, but 15.1 wt% and 10.1 wt% carbohydrates, respectively. In Figure S6 of the supplementary information, the initial polysaccharide composition of the HL1 and HL2 lignins is displayed. Glucose is the major polysaccharide component for both investigated lignins. For both cases, the average molecular weight (*M*_w_) and hydroxyl (OH) content could not be determined because of the poor solubility of these lignins in the respective solvents for GPC and ^31^P-NMR analysis.

Other chemicals such as lye (NaOH, ≥97%) pellets and sulfuric acid (H_2_SO_4_ and 97%) were supplied by VWR Chemicals.

### 3.2. Alkaline Hydrolysis-Acid Precipitation

**Figure 10:**
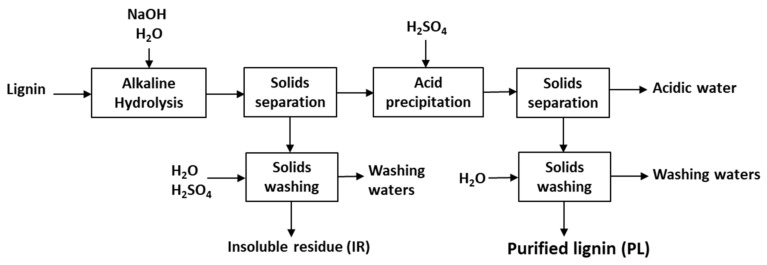
Schematic representation of the alkaline hydrolysis-acid precipitation lab scale protocol.

The alkaline hydrolysis-acid precipitation protocol was investigated for the purification of the HL1 and HL2 hydrolysis lignins. A schematic representation of this protocol can be found in Figure 10. Lignins are hydrolyzed by NaOH at different molar concentrations (from 0.05 M to 1 M) for 3 h at the selected temperature. Afterwards, the mixture is placed in centrifuge vials and centrifuged at 20,000 ×g for 5 min at 25 °C. The insoluble residue (IR) is first washed with acidic water (pH 2) and several times afterwards with abundant ultrapure water at room temperature to adjust its pH close to neutral. To avoid losses of lignin when washing the IR, acidic water is needed in the first step. The liquid phase is slowly acidified with concentrated H_2_SO_4_ until pH 2 to ensure the maximum amount of lignin is precipitated. The mixture is left to settle for at least 30 min before phase separation. Then, it is centrifuged as described above. The precipitate fraction (purified lignin, PL) is washed several times with ultrapure water. Both PL and IR fractions are dried in a vacuum oven at 45 °C for at least 12 h and afterwards kept in airtight containers to avoid moisture adsorption. Each experiment is duplicated.

### 3.3. Acid Hydrolysis

**Figure 11:**
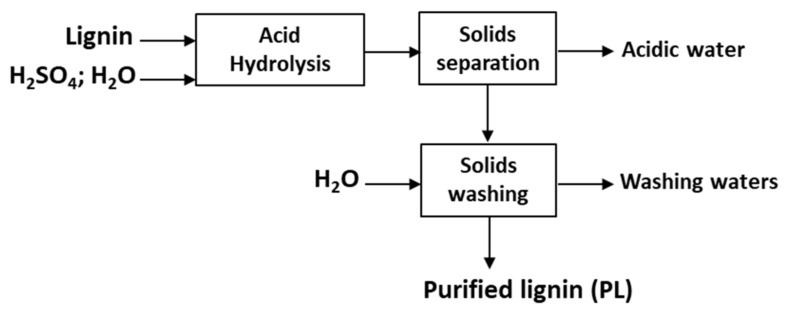
Schematic representation of the acid hydrolysis protocol.

The acid hydrolysis treatment is also investigated in this work for the removal of polysaccharides from lignin. A schematic representation of the one-step acid hydrolysis protocol is presented in Figure 11. Lignins are hydrolyzed by H_2_SO_4_ at different concentrations (0.2–9 M), temperatures (30–200 °C), and time periods (1–22 h). The concentration of lignin for all the acid hydrolysis experiments is kept constant at 10 wt%. Hydrolysis experiments at low temperatures (25–140 °C) are carried out using a heating plate and Ace pressure tubes stirred at 350 rpm. On the other hand, experiments at high temperatures (160–200 °C) are performed for safety reasons in a 100 mL high pressure stirred reactor (Premex, A-line model). The resulting mixtures are centrifuged and the precipitates (purified lignin, PL) are washed with abundant ultrapure water. The materials are dried in a vacuum oven at 45 °C for at least 12 h. Purified lignins are kept in airtight containers to avoid moisture adsorption. The acid hydrolysis experiments are also carried out in duplicates and average yields calculated.

### 3.4. Analytical Methods

The dry matter (d.m.) content in the lignin samples was measured gravimetrically after placing 0.1 g of sample in an aluminum basket, subsequently placed in a ventilated oven, and dried overnight at 105 °C. On the other hand, the ash content is gravimetrically determined after incinerating the sample in a ventilated oven for 4 h at 900 °C. The amount of 0.2 g of dry sample (moisture free) is weighed in an aluminum basket and thereafter placed in the oven.

The standard analysis protocol by the National Renewable Energy Laboratory (NREL) [47] is used for the determination of the carbohydrate content in the lignin samples. The amount of 0.1 g lignin is treated with 1 mL 72% *w*/*w* H_2_SO_4_ solution at 30 °C for 1 h. The mixture is then diluted until 4% H_2_SO_4_ and autoclaved at 121 °C at 1 h. Subsequently, 1 mL of the mixture is mixed with 0.5 mL 400 mM NaOH solution and centrifuged during 10 min at 13,000× g. Finally, the liquid phase is analyzed by High-Performance Anion Exchange Chromatography coupled with a Pulsed Amperometric Detector (HPAEC-PAD), which was supplied by Thermo Scientific (Dionex ICS-5000). The system is equipped with a Dionex CarboPac PA1 column (4 × 250mm) and a Dionex CarboPac PA1 guard column (4 × 50 mm) operated at 25 °C. The mobile phase at a flow rate of 1 mL/min is composed of 250 mM NaOH, 1 M sodium acetate, and ultrapure water in different gradient mixtures. Mannitol, fucose, rhamnose, arabinose, galactose, glucose, xylose, fructose, ribose, fucose, mannitol, galacturonic acid, and glucuronic acid were used as calibration standards in ultrapure Milli Q water with concentrations from 10 mg/L to 500 mg/L. Quality control samples with a specified polysaccharide concentration are also included in the calibration series. The relative standard deviation is lower than 3%.

The average molecular weight (*M*_w_) of the lignin samples is determined by gel permeation chromatography (GPC) in alkaline water (0.1 M NaOH). An in-house GPC method comprised of the following: Loading of 100 µl of sample on Ultrahydrogel 120 (300 mm × 7.8 mm; 6 µm particle size) and Ultrahydrogel 500 (30 mm × 7.8 mm; 10 µm particle size) columns (Waters) coupled in series, with a separation range from 100 g/mol to 400,000 g/mol. An isocratic elution is used with a mobile phase consisting of a solution of 0.1 mol/L NaNO_3_ and acetonitrile 80:20 (*v*/*v*). The pH of the eluent is adjusted to about 10.5 by adding 50% (m/m) NaOH solution. The flow rate is 0.4 mL/min and the column temperature is 40 °C. Eluting compounds are monitored using a UV detector. Sodium polystyrene sulfonate analytical standards with a *M*_w_ range from 208 g/mol to 194,000 g/mol are used for *M*_w_ calibration.

The elemental content of Na and S in the samples is determined by Inductively Coupled Plasma-Atomic Emission Spectroscopy (ICP-AES) after acid digestion. About 0.25 g (dried at 105 °C) is introduced in the digestion tube. After the addition of 5 mL of HNO_3_ and 1 mL of H_2_O_2_, the following digestion is performed: Ramp of 30 min to 200 °C and remain at 200 °C for 210 min (using MW7000 Anton Paar digestion system). After cooling, the sample is further diluted to 50 mL. The digested solution is analyzed with ICP-AES (Thermo Icap 6500 or Agilent Technologies 5100) for the determination of the elements Na and S.

The lignin aliphatic, aromatic, and carboxylic acid hydroxyl contents are determined by ^31^P-NMR following the guidelines suggested in the literature [48] and using cyclohexanol as the internal standard and 2-chloro-4,4,5,5-tetramethyl-1,3-2-dioxaphospholane (TMDP) as the phosphorylating reagent.

The FTIR spectra are recorded in a Bruker spectrometer (Alpha optics) using a Diamond Attenuated Total Reflection (ATR) accessory. The ATR unit is designed as a horizontal crystal with a type of clamping utility to ensure good sample contact of solids. The transmittance (%) is measured from 400 cm^−1^ to 4000 cm^−1^.

## 4. Conclusions

In this study two simple purification strategies, the alkaline hydrolysis-acid precipitation and the acid hydrolysis, were proposed for the removal of residual polysaccharides (10–15% d.m.) covalently linked to hardwood hydrolysis lignins. The two methodologies demonstrated their capability to reduce the polysaccharide content of these hydrolysis lignin samples, which enhances their valorization potential into valuable industrial applications. The alkaline hydrolysis-acid precipitation combining both fractionation and hydrolysis allows the obtaining of a purified lignin fraction with minimal polysaccharide content, which is separated from an alkali-insoluble residue and an acidic-aqueous phase rich in polysaccharides. On the other hand, the acid hydrolysis purification methodology targets the cleavage of the covalent lignin–polysaccharide linkages. The influence of the most relevant process parameters of the studied purification strategies was evaluated and the obtained lignin samples were characterized in terms of polysaccharide content, molecular weight, OH content, and ATR-FTIR analyses. Despite the possibility to operate at ambient temperatures and the minimal polysaccharide content of the purified lignin fractions (< 1% d.m.) obtained after the alkaline-hydrolysis acid precipitation, the lower yields of purified lignin with 19–50% compared to >95% for the acid hydrolysis are an important drawback. The use of lower acid concentrations (0.2 M H_2_SO_4_) during the acid hydrolysis strategy could be compensated by the utilization of higher temperatures (180–200 °C), considering that low polysaccharide lignin samples (0.2–5% d.m.) are obtained under those conditions. Finally, from an industrial and economic point of view, the acid hydrolysis purification strategy using low acid concentrations and high temperatures is more promising compared to the alkaline hydrolysis-acid precipitation, mainly due to the higher yield of purified lignin product. However, further research in terms of capital investments and waste water treatments are needed to accurately evaluate the profitability of both purification strategies.

## Figures and Tables

**Table 1:** Influence of NaOH concentration, temperature, and initial weight % lignin on the OH content of the purified lignin fractions for HL1 and HL2 lignins.

Lignin	*W*_lig_	T (°C)	NaOH (M)	Aliphatic OH	Aromatic OH	Carboxylic Acid	Total OH
				(mmol/g)	(mmol/g)	(mmol/g)	(mmol/g)
HL1	0.03	25	0.1	0.9	3.2	0.3	4.4
	0.03	25	0.5	1.3	3.5	0.3	5.0
	0.03	25	1.0	1.1	3.8	0.3	5.3
	0.03	50	0.1	0.9	3.0	0.3	4.2
	0.01	25	0.1	1.1	3.2	0.4	4.7
HL2	0.03	25	0.1	2.0	2.0	0.2	4.2
	0.03	25	0.5	2.1	2.4	0.3	4.8
	0.03	25	1.0	2.0	2.6	0.3	4.9
	0.03	50	0.1	2.1	2.5	0.3	4.9
	0.01	25	0.1	*na*	*na*	*na*	*na*

*na*: not available (not soluble in the ^31^P-NMR solvent).

**Table 2:** Required input and output for the alkaline hydrolysis-acid precipitation of one metric ton hydrolysis lignin and an estimation of associated costs and revenues.

Variable Costs	HL1	Cost	HL2	Cost	Indicative Unit Cost; Info
Lignin feedstock	1000 kg HL1 845 kg lignin	€254	1000 kg HL2 878 kg lignin	€263	€300/t lignin [42]
Fresh water	33 m^3^	€33	33 m^3^	€33	€1/m^3^; W_Lign_ = 0.03
Lye (50% NaOH)	264 kg	€79	264 kg	€79	€300/t [43]; [NaOH] = 0.1 M
H_2_SO_4_ (96%)	169 kg	€13	169 kg	€13	€75/t [44]; stoichiometric
Waste water treatment	33.4 m^3^	min. €50	33.2 m^3^	min. €50	€1.5/m^3^ (excluding primary treatment) ^a^
Total		€428		€438	
**Revenues**	**HL1**	**Revenue**	**HL2**	**Revenue**	**Indicative unit cost; Info**
Lignin precipitate (LP)	337 kg LP 331 kg lignin	€331	452 kg LP 444 kg lignin	€444	€1000/t lignin content [42]
Lignin residue (LR)	530 kg LR 457 kg lignin	€114	435 kg LR389 kg lignin	€97	€250/t lignin (expected to be of lower value than the respective feedstock)
Total		€446		€541	
**Balance**		**€17**		**€103**	

^a^ Cost estimation for primary waste water treatment is not possible at this stage since it requires further research.

**Table 3:** Required input and output for the alkaline hydrolysis-acid precipitation of one metric ton hydrolysis lignin and an estimation of associated costs and revenues.

Variable Costs	HL 1	Cost	HL 2	Cost	Indicative Unit Cost; Info
Lignin feedstock	1000 kg HL1 845 kg lignin	€254	1000 kg HL2 878 kg lignin	€263	€300/t lignin [42]
Fresh water	10 m^3^	€10	10 m^3^	€10	€1/m^3^; W_Lign_ = 0.10
Lye (50% NaOH)	320 kg	€96	320 kg	€96	€300/t [43] (stoichiometric)
H_2_SO_4_ (96%)	204 kg	€15	204 kg	€15	€75/t [44]; [H_2_SO_4_] = 0.2 M
Natural gas	2.58 MWh ^a^	€39	2.58 MWh ^a^	€39	€15/MWh ^a^ nat. gas [46] (90% boiler efficiency)
Waste water treatment	10.3 m^3^	min. €15	10.5	min. €16	€1.5 EUR/m^3^ (excluding primary treatment) ^b^
Total		€439		€449	
**Revenues**	**HL 1**	**Revenue**	**HL 2**	**Revenue**	**Indicative Unit Cost; Info**
Lignin product	841 kg product 837 kg lignin	€837	873 kg product 869 kg lignin	€869	€1000/t lignin [42]
Total		€837		€869	
**Balance**		**€397**		**€420**	

^a^ Higher heating value; ^b^ Cost estimation for primary waste water treatment is not possible at this stage since it requires further research.

**Table 4:** Characteristics of hydrolysis lignins studied in this work.

Parameter	HL1	HL2
Dry matter content (d.m.; wt%)	97.0	82.3
Ash (wt% of d.m.)	0.4	2.1
Physical state	Powder	Powder
Sodium, Na (wt% of d.m.)	0.01	0.59
Sulfur, S (wt% of d.m.)	0.03	0.29
Carbohydrates (wt% of d.m.)	15.1 ± 0.5	10.1 ± 0.4
Feedstock	Hardwood (mix)	Hardwood (birch)

## Data Availability

The data presented in this study are available in this article and supplementary material.

## Supplementary Materials

The following are available online, Table S1. Characteristic FTIR lignin bands and their assignments according to literature data, Table S2. Modified FTIR lignin bands after removal of polysaccharides and their assignments according to literature data, Figure S1. FTIR spectra of (a) initial HL1 and (b) HL2 lignins and the corresponding purified lignin fractions and insoluble residues after the alkaline hydrolysis-acid precipitation using 0.1 M NaOH (initial *W*_lign_ = 0.03, T = 25 °C), Figure S2. (a) FTIR spectra of the initial HL1 lignin and purified lignin fractions using NaOH at different concentrations (initial *W*_lign_ = 0.03, T = 25 °C) and (b) FTIR spectra of the initial HL2 lignin and purified lignin fractions obtained at different temperatures (0.1 M NaOH, initial *W*_lign_ = 0.03), Figure S3. FTIR spectra of the initial HL1 lignin and samples after the acid hydrolysis using H_2_SO_4_ at different concentrations (T = 30 °C, t = 1 h), Figure S4. FTIR spectra of the initial HL1 lignin and samples after the acid hydrolysis using 2 M H_2_SO_4_ at different temperatures and times, Figure S5. FTIR spectra of the initial HL2 lignin and samples after the acid hydrolysis using 0.2 M H_2_SO_4_ at different temperatures for 1 h, Figure S6. Initial polysaccharide distribution in the HL1 and HL2 lignin samples.
