# Supplementary material for: Strategies for the Removal of Polysaccharides from Biorefinery Lignins: Process Optimization and Techno Economic Evaluation
Source: Molecules. 2021 Jun 1;26(11):3324. doi: 10.3390/molecules26113324 (PMC8197879; doi:10.3390/molecules26113324)
Supplement: Supplementary file 1 [file molecules-26-03324-s001.zip › molecules-1238905-supplementary.pdf]

SUPPLEMENTARY INFORMATION:

Strategies for the Removal of Polysaccharides from Biorefinery Lignins: Process Optimization and Techno Economic Evaluation

Sandra Corderi [1], Tom Renders [2], Kelly Servaes [1], Karolien Vanbroekhoven [1], Tony De Roo[2] and Kathy Elst [1*]

[1] Flemish Institute for Technological Research (VITO), Separation & Conversion Technology, Boeretang 200, 2400 Mol, Belgium
[2] INEOS Phenol Belgium NV, Haven 1930, Geslecht 1, 9130 Beveren, Belgium
* Correspondence: kathy.elst@vito.be

1. FTIR Spectra and Band Assignments

1.1. FTIR Band Assignment

Table S1. Characteristic FTIR lignin bands and their assignments according to literature data.

General lignin bands (cm^{-1})	Assignment	References
3412-3460	OH stretching	[1]
3388	hydroxyl groups in phenolic and aliphatic structures	[2]
2842-3000	C-H stretching of the methyl and methylene group	[1]
2689-2880	C-H stretching methyl group of methoxyl	[1]
2928	C-H stretching	[2]
1420-1603	C=C aromatic skeletal stretching	[1-3]
1323-1327	syringyl ring breathing with C-O stretching	[1,3,4]
1270	C-O of guaiacyl ring	[1,2]
1264	syringyl structures	[2,4]
1223	syringyl structures	[2]
1218	syringyl and guaiacyl ring breathing with C-O stretching	[1,3]
1034-1135	aromatic C-H in plane deformation	[1,3,4]
1030	C-O deformations of secondary alcohols and aliphatic ethers	[1]
915	C-H out-of-plane in positions 2, 5 and 6 of guaiacyl units	[1]
836	aromatic C-H out of plane bending	[3]

Table S2. Modified FTIR lignin bands after removal of polysaccharides and their assignments according to literature data.

Modifications after LCC cleavage (cm^{-1})	Assignment	References
1693 - 1728	C=O stretching in carbohydrates	[3-5]
1269	C=O stretching in carbohydrates	[3,5]
1156	C-O stretching in ester groups in carbohydrates	[4]
1036	C-O stretching in ether bond in carbohydrates	[2]

1.2. Alkaline Hydrolysis – Acid Precipitation

The FTIR spectra of initial HL1 and HL2 lignins, purified lignins and insoluble residues after the alkaline hydrolysis - acid precipitation treatment are shown in Figure S1 (a) and (b) (supplementary information). A reduction is shown for the 1036 and 1156 cm^{-1} bands in the purified lignin fraction of HL1 compared to initial lignin sample or insoluble residue, which has a high carbohydrate content. Regarding the HL2 lignin, the purified lignin fraction shows a decrease in the 1036 and 1693 cm^{-1} bands, which confirmed its lower polysaccharide content compared to the initial sample or insoluble residue.

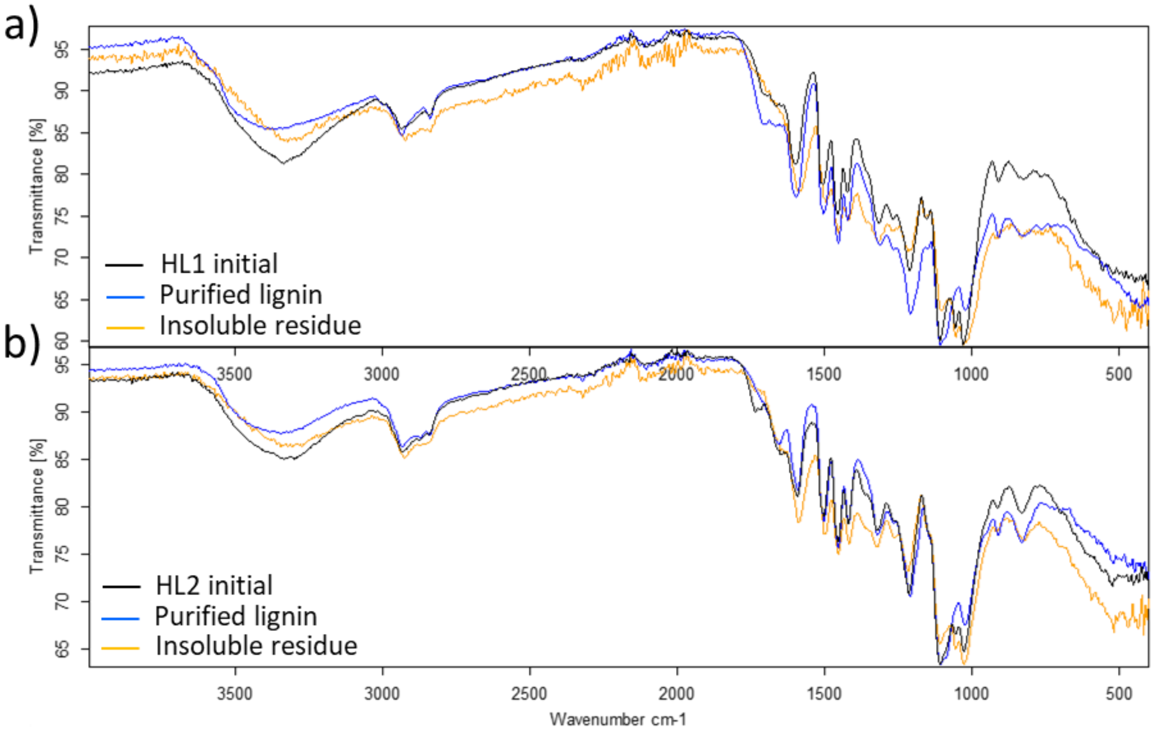

Figure S1. FTIR spectra of (**a**) initial HL1 and (**b**) HL2 lignins and the corresponding purified lignin fractions and insoluble residues after the alkaline hydrolysis - acid precipitation using 0.1 M NaOH (initial W_{lign} = 0.03, T = 25 °C).

No significant differences in the FTIR spectra of purified lignins, obtained under different NaOH concentrations, temperatures or initial mass fraction of lignin, are observed (Figure S2, supplementary information). This could be explained by the high purity of purified lignin fractions, which contain in all cases less than 1 % d.m. polysaccharides.

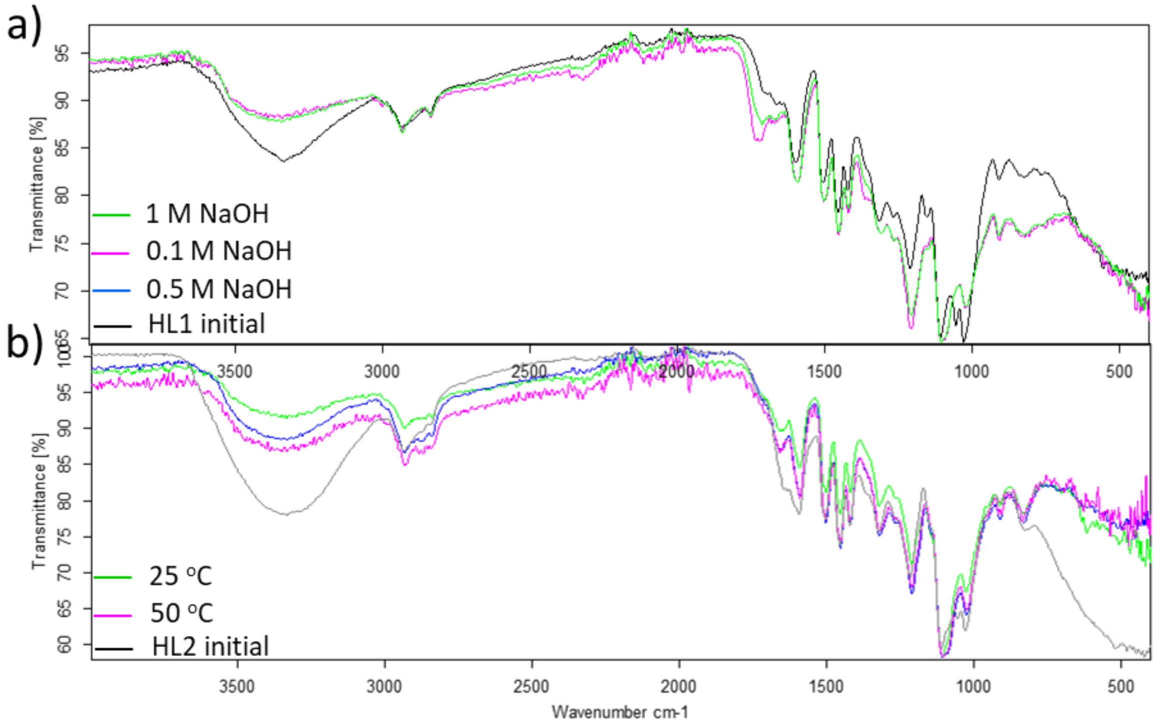

Figure S2. (**a**) FTIR spectra of the initial HL1 lignin and purified lignin fractions using NaOH at different concentrations (initial W_{lign} = 0.03, T = 25 °C). (**b**) FTIR spectra of the initial HL2 lignin and purified lignin fractions obtained at different temperatures (0.1 M NaOH, initial W_{lign} = 0.03).

1.3. Acid Hydrolysis

In Figure S3, it is shown that the increase of acid concentration for HL1 lignin leads to a slight decrease of the 1036 cm^{-1} band, which is in concordance with the reduction of polysaccharide concentration in the samples. The influence of acid hydrolysis temperature and time in the lignin FTIR spectra is observed in Figure S4 for HL1 lignin. The lower the polysaccharide concentration in lignin samples, increasing temperature and time, the smaller the polysaccharide bands at 1036 and 1156 cm^{-1}. The effect acid hydrolysis temperature for low acid concentration experiments (0.2 M H_2SO_4) is represented in the FTIR spectra of Figure S5 for HL2 lignin. As mentioned above, the reduction of the carbohydrate content in the samples by increasing temperature is observed in the modification of the band at 1036 cm^{-1}.

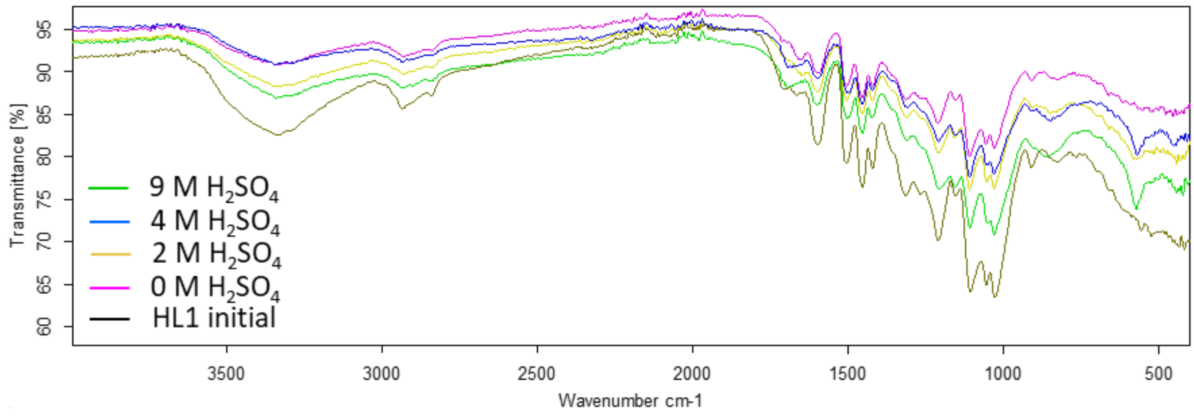

Figure S3. FTIR spectra of the initial HL1 lignin and samples after the acid hydrolysis using H_2SO_4 at different concentrations (T = 30 °C, t = 1 h).

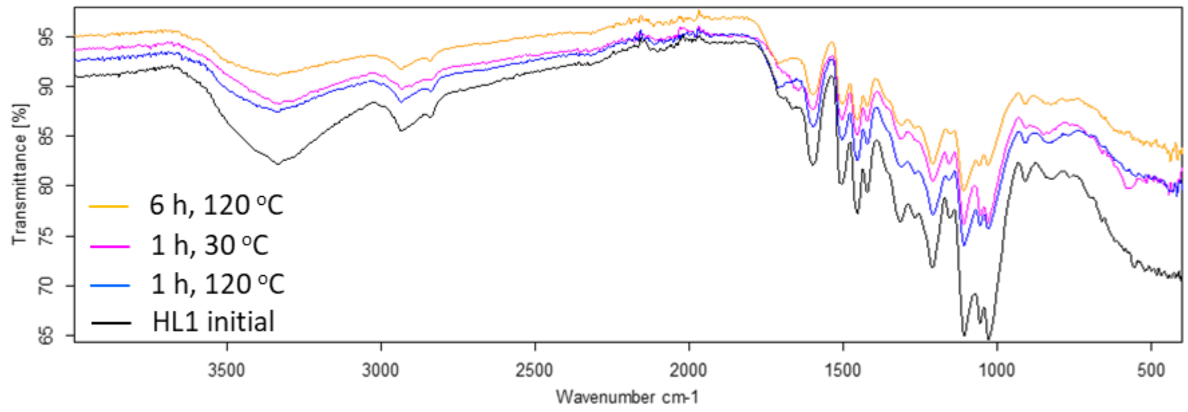

Figure S4. FTIR spectra of the initial HL1 lignin and samples after the acid hydrolysis using 2 M H_2SO_4 at different temperatures and times.

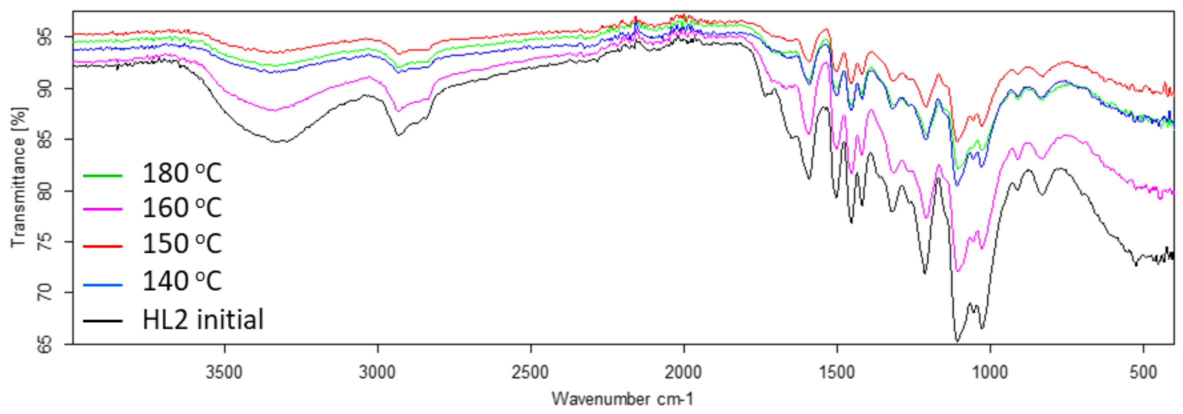

Figure S5. FTIR spectra of the initial HL2 lignin and samples after the acid hydrolysis using 0.2 M H_2SO_4 at different temperatures for 1 hour.

2. Polysaccharide Distribution in HL1 and HL2 Lignins

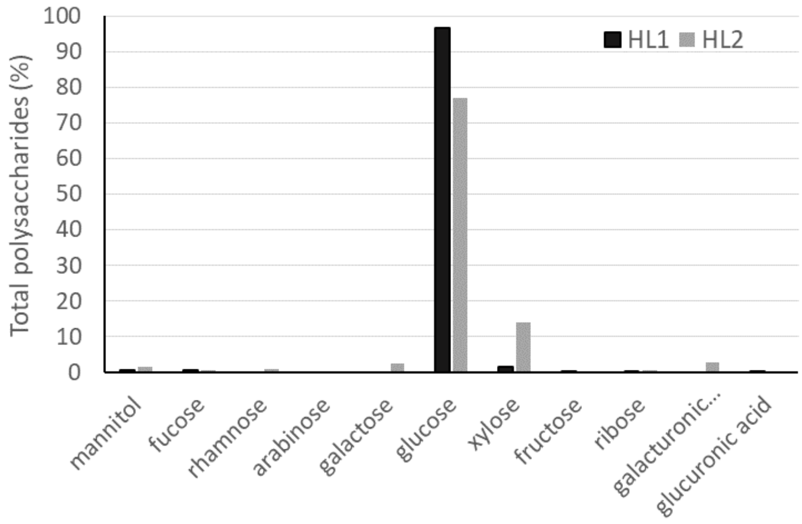

Figure S6. Initial polysaccharide distribution in the HL1 and HL2 lignin samples.

3. References

1. Nunes, K.S.D.; Pardini, L.C. Purification and characterization methods for lignin biomass as a potential precursor for carbon materials. *Cellul. Chem. Technol.* **2019**, *53*, 227-242.
2. Wang, G.H.; Chen, H.Z. Carbohydrate elimination of alkaline-extracted lignin liquor by steam explosion and its methylolation for substitution of phenolic adhesive. *Ind. Crops Prod.* **2014**, *53*, 93-101, doi:10.1016/j.indcrop.2013.12.020.
3. Dai, L.X.; Lu, H.M.; Zhang, L.P. The Purification of Industrial Alkali Lignin with Xylanase. In *Biotechnology, Chemical and Materials Engineering Iii, Pts 1 and 2*, Sung, W.P., Chen, R., Eds.; Advanced Materials Research; 2014; Volume 884-885, pp. 598-602.
4. Liu, Y.Z.; Chen, W.S.; Xia, Q.Q.; Guo, B.T.; Wang, Q.W.; Liu, S.X.; Liu, Y.X.; Li, J.; Yu, H.P. Efficient Cleavage of Lignin-Carbohydrate Complexes and Ultrafast Extraction of Lignin Oligomers from Wood Biomass by Microwave-Assisted Treatment with Deep Eutectic Solvent. *Chemsuschem* **2017**, *10*, 1692-1700, doi:10.1002/cssc.201601795.
5. Singh, R.; Singh, S.; Trimukhe, K.D.; Pandare, K.V.; Bastawade, K.B.; Gokhale, D.V.; Varma, A.J. Lignin-carbohydrate complexes from sugarcane bagasse: Preparation, purification, and characterization. *Carbohydr. Polym.* **2005**, *62*, 57-66, doi:10.1016/j.carbpol.2005.07.011.
